# Viral RNA recognition by LGP2 and MDA5, and activation of signaling through step-by-step conformational changes

**DOI:** 10.1093/nar/gkaa935

**Published:** 2020-11-02

**Authors:** Ivana Duic, Hisashi Tadakuma, Yoshie Harada, Ryo Yamaue, Katashi Deguchi, Yuki Suzuki, Shige H Yoshimura, Hiroki Kato, Kunio Takeyasu, Takashi Fujita

**Affiliations:** Division of Integrated Life Science, Graduate School of Biostudies, Kyoto University, Kyoto 606-8501, Japan; Laboratory of Molecular Genetics, Institute for Frontier Life and Medical Sciences, Kyoto University, Kyoto 606-8397, Japan; Division of Protein Chemistry, Institute for Protein Research, Osaka University, Osaka 565-0871, Japan; School of Life Science and Technology, ShanghaiTech University, Shanghai 201210, China; Division of Protein Chemistry, Institute for Protein Research, Osaka University, Osaka 565-0871, Japan; Division of Integrated Life Science, Graduate School of Biostudies, Kyoto University, Kyoto 606-8501, Japan; Laboratory of Molecular Genetics, Institute for Frontier Life and Medical Sciences, Kyoto University, Kyoto 606-8397, Japan; Division of Integrated Life Science, Graduate School of Biostudies, Kyoto University, Kyoto 606-8501, Japan; Frontier Research Institute for Interdisciplinary Sciences, Tohoku University, Sendai 980-8578, Japan; Division of Integrated Life Science, Graduate School of Biostudies, Kyoto University, Kyoto 606-8501, Japan; Laboratory of Molecular Genetics, Institute for Frontier Life and Medical Sciences, Kyoto University, Kyoto 606-8397, Japan; Institute for Cardiovascular Immunology, University Hospital Bonn, University of Bonn, Bonn 53127, Germany; Division of Integrated Life Science, Graduate School of Biostudies, Kyoto University, Kyoto 606-8501, Japan; Division of Integrated Life Science, Graduate School of Biostudies, Kyoto University, Kyoto 606-8501, Japan; Laboratory of Molecular Genetics, Institute for Frontier Life and Medical Sciences, Kyoto University, Kyoto 606-8397, Japan; Institute for Cardiovascular Immunology, University Hospital Bonn, University of Bonn, Bonn 53127, Germany

## Abstract

Cytoplasmic RIG-I-like receptor (RLR) proteins in mammalian cells recognize viral RNA and initiate an antiviral response that results in IFN-β induction. Melanoma differentiation-associated protein 5 (MDA5) forms fibers along viral dsRNA and propagates an antiviral response via a signaling domain, the tandem CARD. The most enigmatic RLR, laboratory of genetics and physiology (LGP2), lacks the signaling domain but functions in viral sensing through cooperation with MDA5. However, it remains unclear how LGP2 coordinates fiber formation and subsequent MDA5 activation. We utilized biochemical and biophysical approaches to observe fiber formation and the conformation of MDA5. LGP2 facilitated MDA5 fiber assembly. LGP2 was incorporated into the fibers with an average inter-molecular distance of 32 nm, suggesting the formation of hetero-oligomers with MDA5. Furthermore, limited protease digestion revealed that LGP2 induces significant conformational changes on MDA5, promoting exposure of its CARDs. Although the fibers were efficiently dissociated by ATP hydrolysis, MDA5 maintained its active conformation to participate in downstream signaling. Our study demonstrated the coordinated actions of LGP2 and MDA5, where LGP2 acts as an MDA5 nucleator and requisite partner in the conversion of MDA5 to an active conformation. We revealed a mechanistic basis for LGP2-mediated regulation of MDA5 antiviral innate immune responses.

## INTRODUCTION

RIG-I-like receptors (RLRs) are mammalian cytosolic pattern-recognition receptors (PRRs) activated by viral RNA species (1,2). The members of the RLR family are: retinoic acid inducible gene I (RIG-I), melanoma differentiation-associated gene 5 (MDA5), and laboratory of genetics and physiology 2 (LGP2). All of the family members have a highly homologous structure, in particular the central DExD/H box RNA helicase domain. The helicase and the C terminal domain (CTD) are responsible for RNA recognition. RIG-I and MDA5 have N terminal caspase activation and recruitment domain (CARD), which is essential for signal transduction.

Although structurally homologous, RLRs differ in their RNA recognition and signaling capability; however, their commonality is that interaction with non-self RNA induces a conformational change, leading to the exposure of the CARDs. In the presence of ATP, RLR dissociate from non-self RNA, interpreted as possible negative regulation (3,4). The exposed CARDs interact with an adaptor protein, mitochondrial antiviral signaling protein (MAVS) (5). MAVS acts as a signaling platform that facilitates the activation of transcription regulators, including IRF-3 and NFκB, leading to the transcription of the genes encoding type I interferon (IFN) and IFN-inducible genes (5–8).

Although it strongly binds RNA, LGP2 lacks CARDs or any other known signaling domain. LGP2 is present at low levels in uninfected cells but accumulates in response to viral infection (9). It has the ability to recognize various RNAs, irrespective of length or 5′ phosphate ends (10–12). Therefore, it was considered to be a dominant negative regulator (9,13,14). However, analyses of LGP2 -/- animals and cells revealed that it has positive regulatory function for activation by RIG-I and MDA5 (15).

The function of LGP2 in the immune response is controversial due to different reports depending on the experimental approaches. Growing evidence suggests a positive role of LGP2 in MDA5 antiviral signaling. For example, LGP2-associated EMCV RNA was found to act as a physiological agonist of MDA5 (16).

In this report, we investigated the role of LGP2 in MDA5-induced antiviral signaling. We focused on viral RNA recognition by MDA5, the involvement of ATP and its hydrolysis, and conformational changes of MDA5 through these events.

## MATERIALS AND METHODS

### Cell culture and plasmids

HEK293T cells were maintained in Dulbecco's modified Eagle's Medium (DMEM) with 10% fetal bovine serum (FBS) and penicillin/streptomycin (Nacalai Tesque, Japan).

p-125 Luc and p-RL-tk were described previously (1). pEF-BOS-FLAG-MDA5 and pEF-BOS-FLAG-LGP2 were obtained by subcloning cDNA into the empty pEF-BOS vector with the oligonucleotides for the N-terminal 2x FLAG-tag.

### Preparation of BPEVdsRNA

The genomic 14 k bp linear dsRNA of bell pepper endornavirus (BPEV) was prepared as follows. The green peppers (*Capiscum annuum;* Kyosozu strain) were crushed using a low-speed compression juicer. The juice was fractionated into nuclear, organelle, vesicular and cytosolic fractions. The RNA from the organelle fraction was extracted by phenol-chloroform. After treating the sample with DNase 1 (Roche), total RNA was extracted with phenol and precipitated with ethanol. This RNA was further purified by agarose gel electrophoresis and recovered by the GENECLEAN II Kit (MP Biomedicals). The quality and purity of the dsRNA was confirmed by agarose gel electrophoresis and AFM.

### Luciferase assay

HEK293T cells were transfected with p-125 Luc, p-RL-tk, pEF-BOS-MDA5 or with addition of pEF-BOS-LGP2 using linear polyethyleneimine (PEI) under standard conditions. After 24 hours, cells were further transfected with poly(I:C), BPEVdsRNA using PEI or infected by EMVC. The Dual-Luciferase Reporter Assay System was used following the manufacturer's instructions (Promega).

### Production and purification of recombinant RLR proteins

GST-Flag MDA5 was produced using the Bac-to-Bac Baculovirus Expression System (Invitrogen, Life Technologies). The protein was expressed as a GST fusion protein in High Five insect cells and purified using Glutathione Sepharose 4B (GE Healthcare). The GST tag was removed by AcTeV protease (Invitrogen). Coexisting nucleic acids were removed by Q Sepharose HP (GE Healthcare). The final protein conformation was examined by AFM.

6xHis-Flag LGP2 was produced using the Baculovirus Expression System. The protein was expressed as an N-terminal 6xHis tag fusion protein in High Five insect cells. 6xHis-Flag LGP2 was bound to Ni Sepharose 6 Fast Flow (GE Healthcare) and eluted in elution buffer containing 50 mM Tris–HCl (pH 8.0), 150 mM NaCl, 1.5 mM DTT and 500 mM imidazole. Imidazole was removed by PD-10 Desalting Column (GE healthcare). LGP2 K30G was produced similarly.

### ATPase assay

The reaction mixture (20 μl) contained BPEV dsRNA (500 ng; 10 nM), MDA5 (2 μg; 750 nM) and LGP2 (800 ng; 533 nM) in buffer A (20 mM Tris–HCl pH 7.5, 1.5 mM MgCl_2_, 1.5 mM DTT, in 20 μl). Where indicated, less LGP2 (200 or 400 ng) was included. The mixtures were incubated at room temperature for 10 min. ATP was added at a final concentration of 1 mM and the mixture was incubated at 37°C for 30 min. Free phosphate generated was quantified by BIOMOL Green (Enzo Life Science). Absorbance was measured using a microplate reader 680 at a range of 630–850 nm (BIO-RAD).

### Size exclusion chromatography (SEC)—Sepharose 4B

SEC was used to detect protein size shifts. The 2.4-ml column was assembled using a glass Pasteur pipette with a short capillary tip and cotton ball at the bottom. Columns were sterilized and filled with Sepharose 4B (GE Healthcare). Prior to adding the sample, the column was washed and equilibrated with 5 ml (in total) of buffer B (50 mM Tris, 150 mM NaCl, 1.5 mM DTT and 5 mM MgCl_2_). Typically, samples contained 3.4 μM MDA5, 125 nM dsRNA and 5 mM ATP. After the sample was loaded, 100-μl fractions were collected. Fractions were than analyzed by immunoblotting using anti-Flag antibody or condensed by precipitation by acetone before immunoblotting when the sample was fractionated twice.

### MDA5/poly(I:C) complex formation and fractionation after its dissociation

The complex of recombinant MDA5 (3.4 μM) and poly(I:C) (30 μg) was formed by incubation in buffer B. The complex was isolated by SEC by Sepharose 4B. Pooled fractions containing MDA5/poly(I:C) complexes were treated with 5 mM ATP or 5 mM ATP + 2.5 μM LGP2. Samples were incubated at 37°C for 30 min and then ultra-centrifuged at 300 000 × g for 10 min (Beckman Coulter, Optima MAX-XP, TLA 120.2). Samples were mixed with native sample buffer (0.25 M Tris–HCl, pH 6.8, 40% glycerol, 0.005% bromophenol blue) and then subjected to 6% Native-PAGE (without SDS). Buffers for anode and cathode chambers were 25 mM Tris–HCl, pH 6.8, 192 mM glycine, and the same buffer supplemented with 1% DOC, respectively. The gel was pre-run for 60 min at 40 mA, the samples were electrophorized for 6 h with constant 60 V at 4°C. MDA5 was visualized by immunoblotting (anti-MDA5-CTD antibody).

### Limited trypsin digestion

MDA5 or MDA5/LGP2 alone or complexed with poly(I:C) (at 1:1 mass ratio) in the absence or presence of 2 mM AMP-PNP or 2 mM ATP as 25-μg samples were treated with 35 ng of Trypsin from bovine pancreas (TPCK treated: Sigma-Aldrich). At the indicated time intervals 10-μl samples were removed from the reaction, which was stopped by adding SDS-sample buffer and heating at 95°C for 5 min. Samples were subjected to SDS-PAGE and silver stained using Sil-Best Stain One (Nacalai Tesque, Japan) following the manufacturer's instructions.

### Peptide sequencing

N-terminal amino acid sequence of trypsin-digested MDA5 was determined by Japan Institute of Leather Research.

### Antibodies and antibody labeling

Anti-Flag and anti-GFP were purchased from Sigma and Santa Cruz Biotechnology, respectively. The anti-human LGP2 antibody was generated by immunizing rabbits with a synthetic peptide corresponding to amino acids 535–553 of human LGP2 and affinity purified by using antigen peptide, as described previously (17).

Antibody labeling: anti-LGP2 and anti-GFP were conjugated with Qdot 655 nanocrystals using the SiteClick™ Qdot™ 655 Antibody Labeling Kit following the manufacturer's instructions (Invitrogen, Thermo Fischer Scientific, Life Technologies Corporation, USA).

### AFM sample preparation

Purified recombinant proteins and BPEVdsRNA were diluted in buffer C (5 mM HEPES–NaOH pH 7.5, 50 mM NaCl, 5 mM MgCl_2_, 150 nM MDA5, 8.4 nM dsRNA and 30 nM LGP2). Mixtures were incubated at 37°C. Then, RNA/protein samples were fixed with 0.05% glutaraldehyde (Nacalai Tesque, Japan), placed on 10 mM Spermidine treated mica for adhesion and dried with nitrogen gas before imaging.

### AFM imaging

AFM imaging was performed using the Multimode AFM Nanoscope^®^ IIIa controller and a J scanner (Bruker, Veeco, Digital Instruments). The AFM machine was operated in tapping mode at a scanning rate of 1–2 Hz in air at room temperature using silicon rectangular micro cantilevers with sharpened tetrahedral tips (OMCL-AC160TS-C3, Olympus Corporation, Tokyo, Japan). The cantilevers had a spring constant of 26 N/m and a resonance frequency of 300 (±100) kHz. Images were taken from height data and flattened in the NanoScope Analysis (v. 5.31 rl, Digital Instruments).

### AFM image analysis

The flattened SPM images were analyzed using NanoScope Analysis (v. 5.31 rl, Digital Instruments and 1.40, Bruker). Section analyses were performed using the software by drawing a line at the point of interest and converting the corresponding curve graph data into a text file.

### High-speed AFM

The dynamics of MDA5 molecules were imaged using a high-speed AFM system (Nano Live Vision, Research Institute of Biomolecules Metrology Co., Tsukuba, Japan) with a custom-made piezo scanner, the resonance frequencies of which are *xy* 30 kHz and *z* 600 kHz. Small silicon nitride cantilevers were used (BL-AC10EGS-A2 cantilevers; Olympus Co., Tokyo, Japan). Their resonant frequencies in water were ∼600 kHz and the spring constants in water were ∼0.1 N/m. Each cantilever had an electron beam-deposited (EBD) probe.

A 2-μl droplet of MDA5 solution (0.5 ng/μl) was deposited onto the surface of freshly cleaved mica (1.5 mm in diameter). After incubation for 1 min at room temperature, the sample was gently rinsed several times with buffer C (20 mM Tris–HCl (pH 7.5), 10 mM MgCl_2_ and 1 mM EDTA) to remove unabsorbed molecules. High-speed AFM imaging in tapping mode was performed in the same buffer solution. The 192 × 144-pixel images were obtained at a scan rate of 2.0–5.0 frame/s and analyzed using the ImageJ software (National Institute of Health (NIH), Bethesda, MD, USA, http://rsbweb.nih.gov/ij/).

## RESULTS

### LGP2 increases IFN-β promoter activity through MDA5 upon stimulation by Poly (I:C), EMCV or BPEV dsRNA

**Figure 1.**
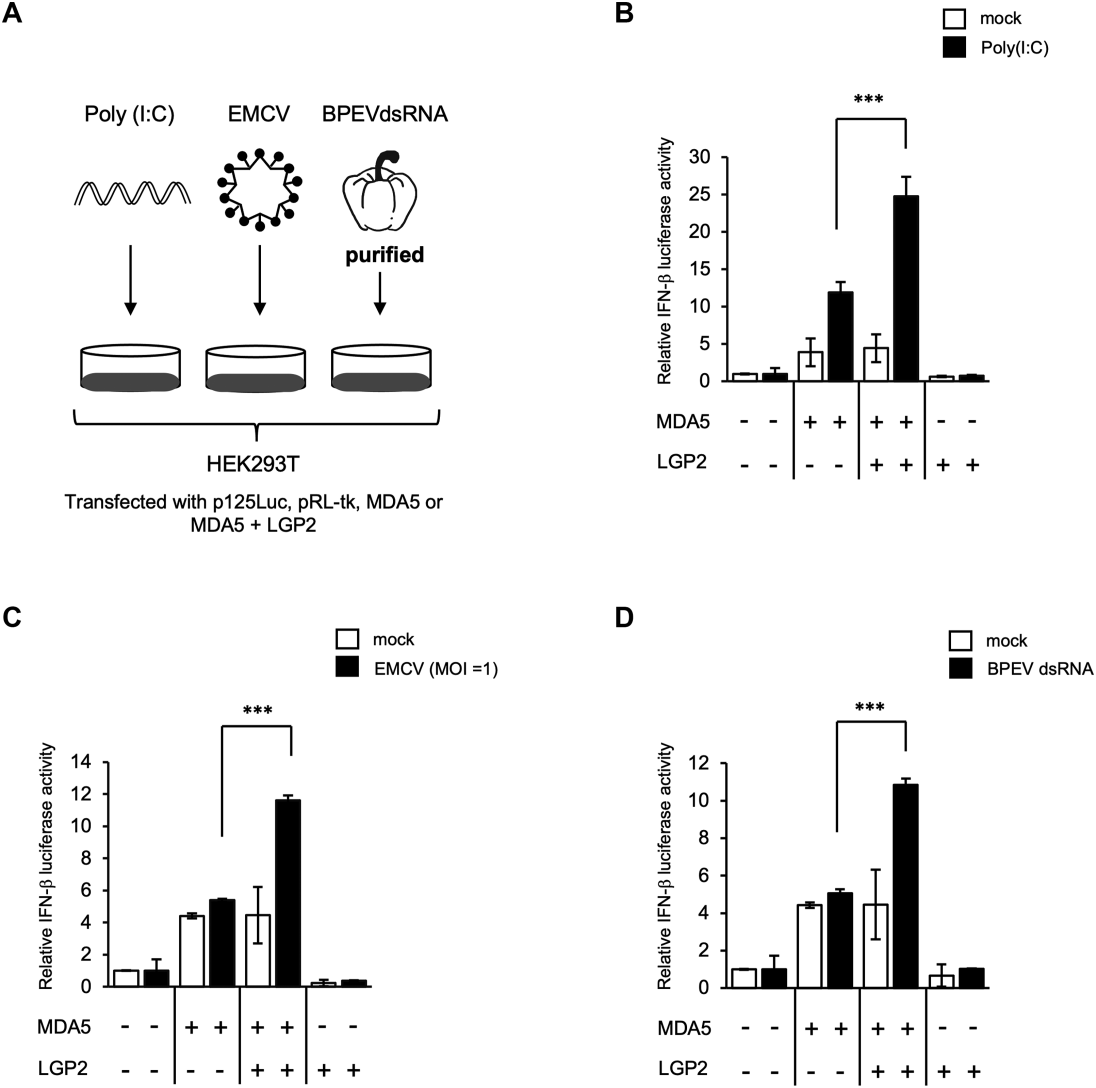
LGP2 increases IFN-β promoter activity through MDA5 in response to poly(I:C), EMCV or BPEVdsRNA. (**A**) Experimental design to analyze IFN-β promoter activity in HEK293T cells in response to different stimuli. (B–D) HEK293T cells in 12-well plate were transfected with a reporter plasmid containing the luciferase gene under the control of the IFN-β promoter (p-125Luc, 1 μg), Renilla luciferase construct (pRL-tk, 40 ng) as an internal control, 25 ng of plasmid expressing MDA5 and 4 ng of plasmid expressing LGP2. Twenty-four hours after transfection, cells were mock treated or (**B**) transfected with 3 μg of poly(I:C), (**C**) infected with EMCV (MOI = 1), and (**D**) transfected with 300 ng of BPEVdsRNA. After 8 h, cells were subjected to the dual-luciferase assay. Data represent relative firefly luciferase activity normalized to Renilla luciferase activity in one of at least three independent experiments, and error bars indicate ± S.D. *** *P* <0.001, * *P* <0.05, unpaired Student's t test.

To investigate the effects of LGP2 on IFN-β induction upon stimulation by different RNA species, we used synthetic RNA poly(I:C), virus (EMCV) and BPEVdsRNA extracted from bell pepper (18) to stimulate MDA5. HEK293T cells were stimulated as described in (Figure 1A). Overexpression of MDA5 augmented the basal promoter activity of IFN-β; however, it was further up-regulated upon poly(I:C) transfection (Figure 1B). Co-expression of LGP2 significantly increased IFN-β promoter activity. These results are consistent with previous reports (19,20). No significant induction of the IFN-β promoter was observed upon EMCV infection in cells expressing MDA5 alone (Figure 1C), whereas clear IFN-β induction was observed in cells expressing LGP2 and MDA5. Similar results were obtained when cells were stimulated by transfection with BPEVdsRNA (Figure 1D). The expression of LGP2 alone failed to activate the IFN-β promoter by either of the stimuli, demonstrating that MDA5 is essential for induction.

### LGP2 increases fiber formation by MDA5

**Figure 2.**
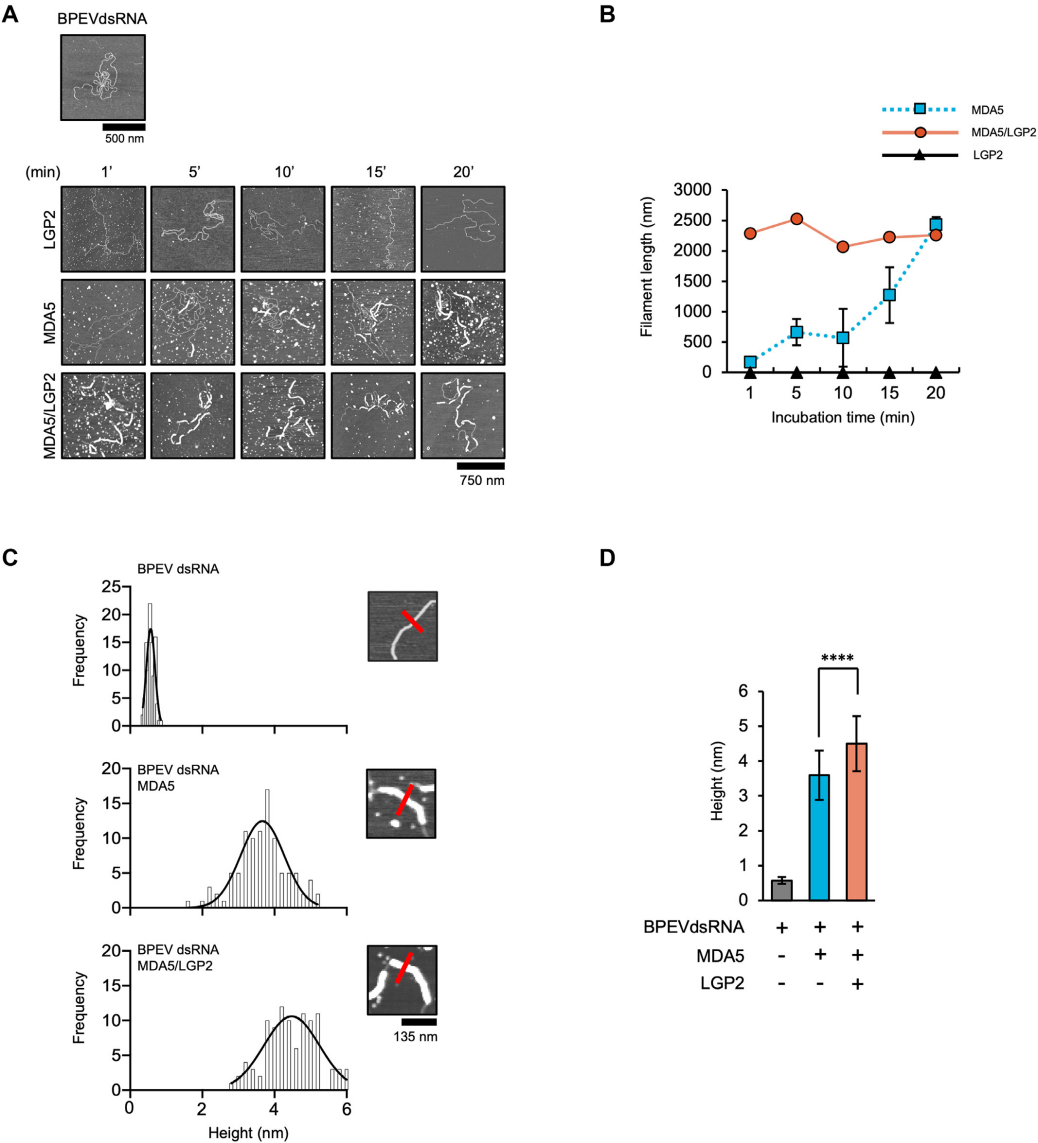
LGP2 increases fiber formation by MDA5. (**A**) AFM images of the time course of binding of different proteins with BPEVdsRNA. AFM image of BPEVdsRNA (top). BPEVdsRNA was mixed with the proteins indicated on the left and observed at indicated time points by AFM. (**B**) The average fiber length measured from at least three independent experiments. Error bars: ± S.D. MDA5 fiber formation. (**C**) Height quantification of BPEVdsRNA alone (top) and fibers formed by MDA5 (middle) or MDA5/LGP2 (bottom). (**D**) Average of BPEVdsRNA height (gray), MDA5 fiber height in the absence (blue) and presence (red) of LGP2, measured from 100 sections. Error bars: ± S.D. **** *P* < 0.0001, unpaired Student's *t*-test.

Electron microscopy revealed that MDA5 binds to long dsRNA by forming fiber-like polymers in which monomers are stacked head-to-tail (4,21,22). LGP2 was previously reported to regulate MDA5 fiber assembly (23). However, the mechanistic basis and detailed ATP roles remained unknown. We utilized AFM, that has been known for visualization of various protein and nucleo-protein complexes (24–26), to gain insight into the MDA5 fiber formation in the presence of LGP2 (Figure 2). LGP2 alone did not form fibers with dsRNA. MDA5 formed fibers in a time-dependent manner. The fiber formation started after 1–5 min of incubation and continued to elongate to reach 2.5 μm in length, covering 70% of the BPEVdsRNA after 20 min (Figure 2A, B). In contrast, in the presence of LGP2, MDA5 fibers formed rapidly, reaching the same length and covering 70% of BPEVdsRNA in less than 1 minute. These results confirmed that LGP2 assisted fiber formation by MDA5.

**Figure 3.**
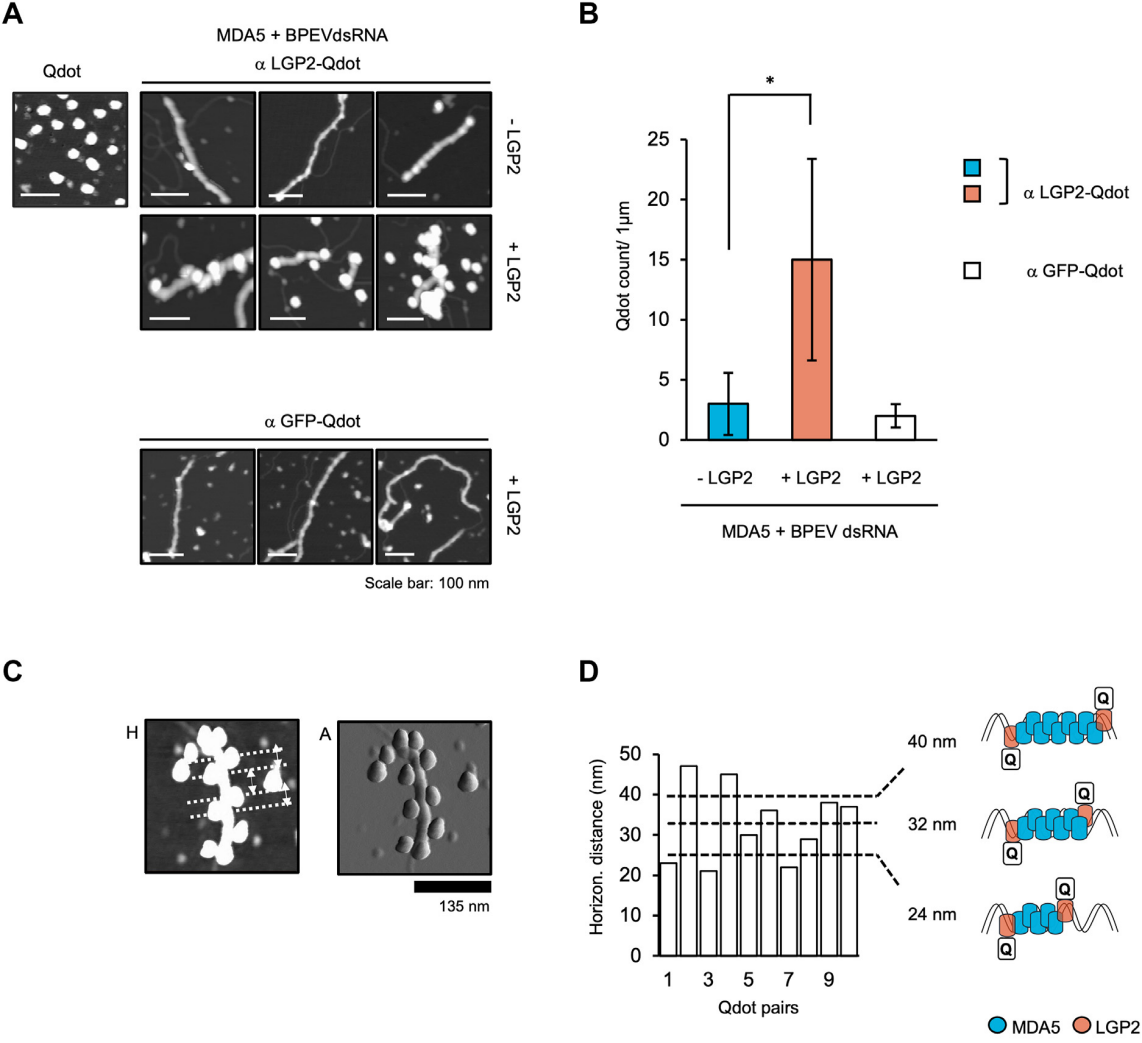
Incorporation of LGP2 into MDA5 fiber. (**A**) AFM images of Qdot (upper left), and MDA5/BPEVdsRNA fiber with or without LGP2 probed with αLGP2-Qdot or αGFP-Qdot as indicated. (**B**) Quantification of average number of Qdots/1 μm of MDA5 fiber. Values are the average of four Qdot counts in 1 μm of MDA5 fiber. Error bars: ±S.D. * *P* < 0.05, unpaired Student's *t*-test. (**C**) Representative AFM image of distance measurements between αLGP2-Qdot attached to the fiber; *H* = height, *A* = amplitude. (**D**) Distances of 10 Qdot pairs (left). Schematic model of fiber composed of MDA5 and LGP2 (right).

To clarify the mechanism of fiber formation by MDA5 and LGP2, we investigated the recruitment of LGP2 within the MDA5-dsRNA fibers. First, the height of the fibers formed in the presence or absence of LGP2 was measured as described in Figure 2C. The height of BPEVdsRNA alone was 0.57 nm on average. Quantification of the average height revealed that MDA5-dsRNA formed 3.6 nm height fibers, whereas in the presence of LGP2, the fiber height was 4.5 nm (Figure 2D). This suggested that the fiber structure changed in the presence of LGP2 due to the incorporation of LGP2. To investigate this further and to determine the localization of LGP2, we used a specific antibody against LGP2 tagged with quantum dot (Qdot655) (Figure 3A). The specificity of the tagged antibody, termed αLGP2-Qdot, was confirmed (Supplementary Figure S1). MDA5 fibers with or without LGP2 were probed with αLGP2-Qdot or control αGFP-Qdot (Figure 3A). αLGP2-Qdot reacted with the fibers formed by MDA5 and LGP2, but not with those without LGP2. Similarly, αGFP-Qdot did not react with the fibers formed in the presence of LGP2. Quantification of Qdots revealed 15 LGP2 per 1 μm fibers on average (Figure 3B). Of note, LGP2 was mostly positioned on alternating sides of the fiber (Figure 3A, C). Next, we measured distances between Qdots (Figure 3C). The average horizontal distance between two Qdots was 32.8 nm (±S.D. 8 nm) (Figure 3D). A previous publication stated that 1 molecule of MDA5 occupies 14–15 bp of dsRNA (27). Taking this (4.7 nm for stretched 15 bp dsRNA) and the range of inter-distance between Qdots (24.8–40.8 = 32.8 ±8 nm) into account, we speculated that one LGP2 molecule exists every 5–9 MDA5 molecules. These results strongly suggested that LGP2 increases MDA5 fiber formation by periodic incorporation into the fibers.

### MDA5 fiber turnover by LGP2 and ATP hydrolysis

**Figure 4.**
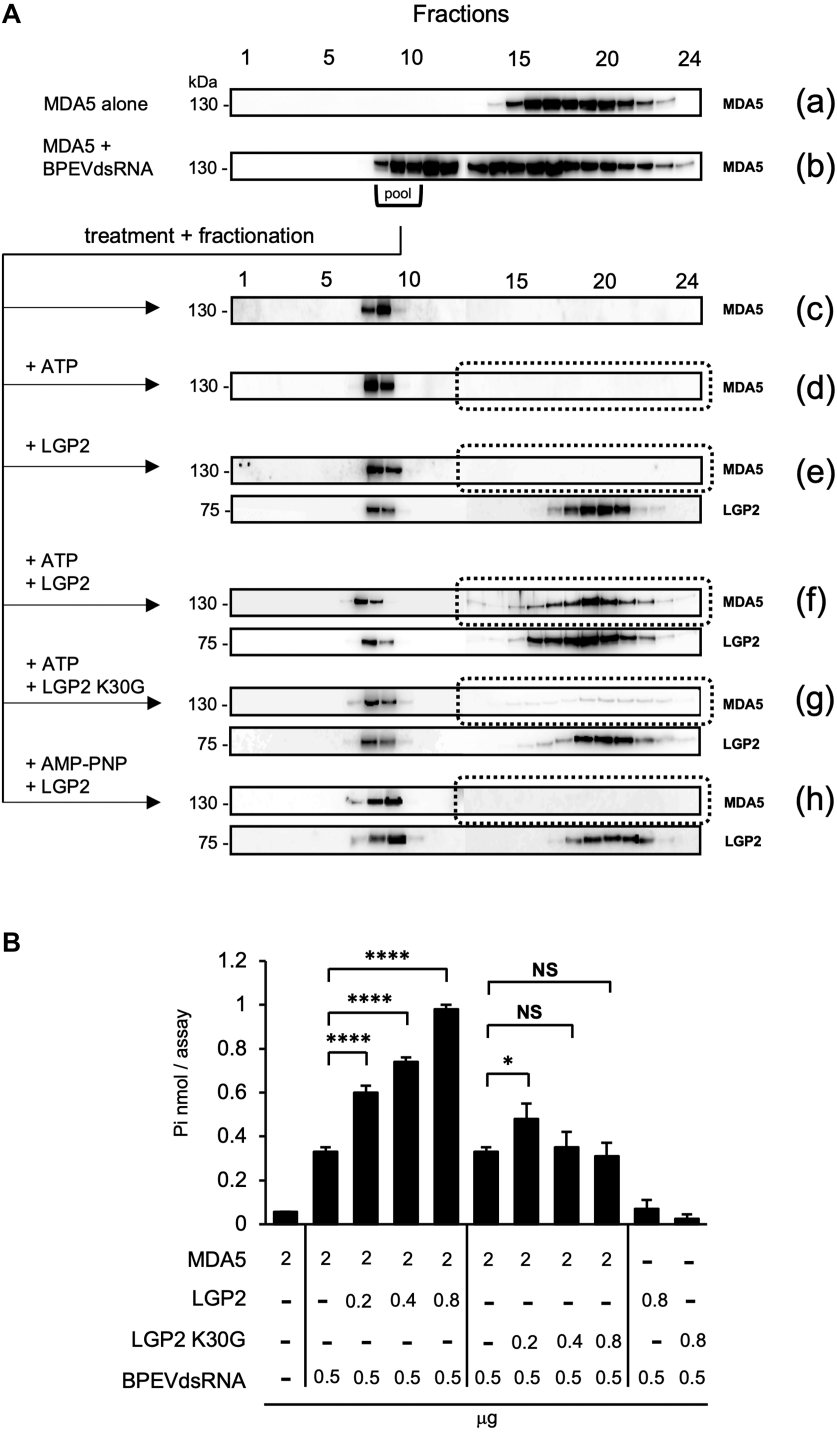
MDA5 fiber turnover by LGP2 and ATP hydrolysis. (**A**) SEC fractionation of free protein and dsRNA/protein complexes. Fractionation of MDA5 (a) and the reaction mixture of MDA5 and BPEVdsRNA (b) by Sepharose 4B. The high-molecular weight fractions (fractions 8–10 of b) were pooled as MDA5/BPEVdsRNA fibers. The fibers were re-chromatographed without incubation (c) or after incubation with ATP (d), LGP2 (e), ATP+LGP2 (f), ATP+LGP2 K30G (g), AMP-PNP+LGP2 (h). Samples were analyzed by immunoblotting (IB) using anti-Flag antibody. (**B**) ATPase assay. Recombinant proteins were analyzed for ATPase activity in the presence or absence of dsRNA (Materials and Methods). Values are the average of 3 independent experiments. Error bars: ±S.D. ****P <0.0001, *P <0.05, NS = *P* > 0.05, unpaired Student's *t*-test.

It was previously reported that MDA5 fibers dissociate in the presence of ATP through its hydrolysis (3,4). We next investigated MDA5 fiber dissociation under different conditions (Figure 4A). First, MDA5 and BPEVdsRNA were incubated, and the fibers were isolated by size exclusion chromatography (SEC) by Sepharose 4B. The isolated fibers were stable and did not generate free MDA5 spontaneously, as confirmed by re-chromatography (c). EM observation revealed that MDA5 fibers dissociate after incubation with ATP (3). However, fiber dissociation by incubation with ATP alone was below the detectable level by SEC (d). This discrepancy may have been due to the lower concentration of MDA5 and dsRNA used in EM observation, therefore lower than the Kd value of MDA5 to dsRNA (Materials and Methods). We estimated molar concentration of MDA5 in cells (Supplementary Figure S2). The result suggested that cellular concentration of MDA5 (2.6 μM) is closer to SEC (3.4 μM) rather than AFM observation (150 nM). These results suggest that SEC condition is physiologically relevant. Incubation of the fibers with LGP2 alone did not generate free MDA5 (e). In contrast, incubation with LGP2 in the presence of ATP partially dissociated the fibers into free MDA5 (f), suggesting that dissociation was markedly increased by LGP2. Interestingly, dissociation by LGP2 K30G, an ATP binding site mutant, was negligible (g). To further investigate the effects of ATP binding and its hydrolysis, we used AMP–PNP, non-hydrolysable ATP analog. Incubation with AMP-PNP/LGP2 failed to dissociate the fibers (h), suggesting LGP2 and ATP hydrolysis are both involved in the efficient dissociation.

Next, we investigated the effects of dsRNA and LGP2 on ATPase activity (Figure 4B). MDA5 exhibited dsRNA-dependent ATPase activity. The addition of LGP2 to the MDA5/dsRNA mixture markedly increased the level of ATPase activity. Addition of LGP2 K30G did not promote ATPase activity. LGP2 alone exhibited modest levels of ATPase activity. These results suggested that the incorporation of LGP2 into MDA5/dsRNA complex promoted the ATP hydrolysis and that the intrinsic ATPase of LGP2 largely contributes to it.

### LGP2 regulates molecular conformation of MDA5

**Figure 5.**
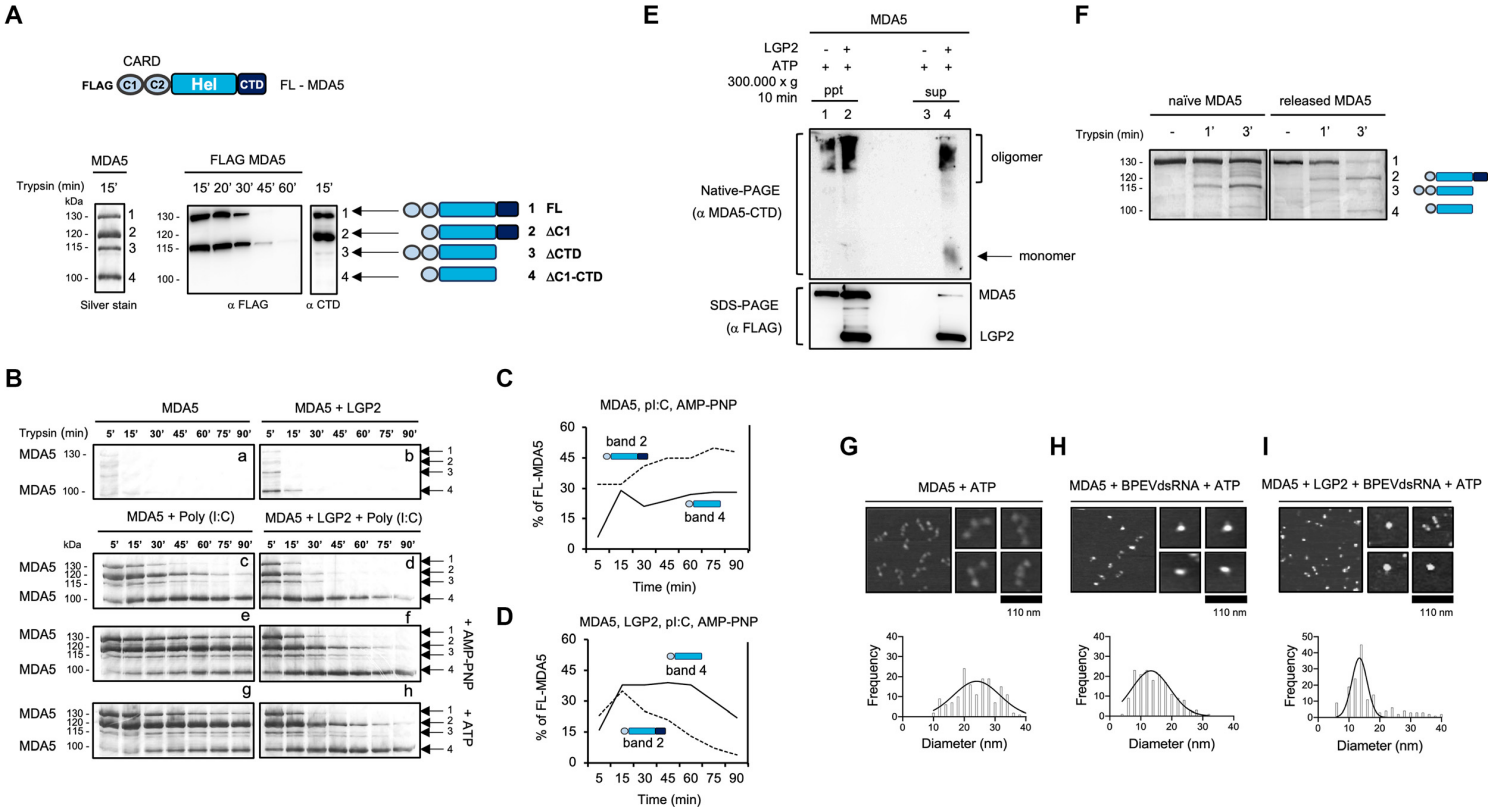
LGP2 regulates molecular conformation of MDA5. (**A**) Schematic representation of MDA5 domain structure (top). Limited trypsin digestion of MDA5 yields 4 major cleavage fragments (silver staining). FLAG-tagged MDA5 was produced in HEK293T cells and digested with trypsin, followed by immunoblotting with anti-FLAG or anti-CTD antibodies. Identification of bands 1–4 is shown. (**B**) Time course of trypsin digestion (TPCK trypsin 35 ng) of recombinant proteins. The indicated recombinant proteins in the presence or absence of poly(I:C), AMP-PNP or ATP were treated with trypsin. The digestion was terminated by adding SDS sample buffer and subjected to SDS-PAGE, followed by silver staining. (**C**) Densitometry of bands 2 and 4 generated by the digestion of MDA5, poly(I:C) and AMP-PNP. (**D**) Densitometry of bands 2 and 4 generated by the digestion of MDA5, LGP2, poly(I:C) and AMP-PNP normalized to non-digested FL-MDA5 as 100%. (**E**) Native PAGE analysis of poly(I:C)/MDA5 complex. MDA5 was mixed with poly(I:C) and the complex was isolated by SEC as in Figure 4Ab. The complex was incubated with ATP in the presence or absence of LGP2. The mixture was fractionated by centrifugation into poly(I:C)-bound complex (ppt) and released proteins (sup). The fractions were analyzed by native (top) and SDS (bottom) PAGE followed by immunoblotting with anti-MDA5 antibody (top) and-Flag antibody (bottom). (**F**) Trypsin digestion of MDA5. Naïve recombinant MDA5 and MDA5 recovered by dissociation from poly(I:C) (lane 4 sample of Fig. 5E) were analyzed by limited trypsin digestion as in (B). Positions of full length MDA5 (1) and its digestion products (2–4) are shown in the right. (**G–I**) AFM images of MDA5 protein under different conditions. Wild type MDA5 protein was incubated with reaction buffer (G) or with dsRNA (H) or with dsRNA and LGP2 (I), in the presence of 1 mM ATP for 30 min and was observed by AFM. Left: 300 × 300 nm^2^ images, Right: 75 × 75-nm^2^ images. From these images, MDA5 monomers (objects with total volume of 300 ± 30 nm^3^) were selected and quantified for diameter (bottom).

The exposure of CARDs of MDA5 and RIG-I was suggested to be the basis for MAVS signaling. To further investigate the conformational change of MDA5 related to CARDs exposure, we used limited digestion by trypsin as a tool for structural analysis. Limited digestion of purified recombinant MDA5 resulted in 4 bands on silver staining (Figure 5A, left). To identify these bands, we overexpressed full-length MDA5 (FL-MDA5) with FLAG tag at the N-terminus in HEK239T cells and digested the cell lysate. Band 1 was identified as full-length MDA5. As bands 2 and 4 did not react with anti-FLAG, these lacked the N-terminus. Similarly, bands 3 and 4 did not react with anti-CTD, suggesting a lack of the CTD in these fragments. We determined N-terminus of band 4 by sequencing (Supplementary Figure S3) and revealed that it lacks CARD1 (C1). From molecular size, these bands were identified as FL, ΔC1, ΔCTD and ΔC1-CTD, respectively (Figure 5A). Thus, bands 2 and 4 are a hallmark of the open (digested) CARD1. Next, we investigated how ATP and/or LGP2 affect the digestion pattern corresponding to MDA5 conformational changes. Recombinant proteins, poly(I:C) and ATP or AMP-PNP were incubated in combination and digested with trypsin. As LGP2 has a smaller molecular size (75 kDa) than band 4, its presence did not interfere with detection of digested MDA5. In the absence of poly(I:C), MDA5 was degraded rapidly and bands 1–4 were barely detectable (Figure 5B, a), suggesting its open conformation observed by AFM (28). Under conditions in which free MDA5 is degraded slowly, band 3 was dominantly detected, but band 2 was barely detectable, suggesting that free MDA5 exposes its CTD but not its CARDs (Supplementary Figure S5A, left). In the presence of MDA5 and poly(I:C), band 4 was detectable even after 90 min of digestion, suggesting that MDA5 helicase is tightly bound to the poly(I:C), but the CARD1 and CTD are exposed (Figure 5B, c). In the presence of poly(I:C) and LGP2, MDA5 became marginally sensitized to digestion (Figure 5B, d), suggesting increased exposure of the CARD1 and CTD. As expected from the observation that AMP-PNP strongly facilitates dsRNA/MDA5 interactions (27), the addition of AMP-PNP conferred resistance to trypsin digestion (compare Figure 5B, c, d with e, f). We quantified the band intensity from Figure 5B, e, f normalized to the non-digested FL-MDA5 as 100% (Supplementary Figure S4). When the fibers composed of MDA5, poly(I:C) and AMP-PNP were digested with trypsin, band 2 was dominantly detected and its intensity increased over time (Figure 5C). In contrast, the intensity of band 4 increased gradually as the digestion proceeded. This suggested that CARD1 was partially exposed under these conditions. When LGP2 was present in the fibers (Figure 5D), band 4 was detected after 5 min of digestion and it became the dominant species as the digestion proceeded. Simultaneously, the intensity of band 2 markedly decreased. These results suggested that the presence of LGP2 in the fibers promoted full exposure of CARD1.

Of note, when ATP was included instead of AMP-PNP (Figure 5B, g, h), the digestion pattern was almost identical to that with AMP-PNP (Figure 5B, e, f). Because incubation of the MDA5/LGP2/dsRNA complex with ATP, but not AMP-PNP, resulted in dissociation of the complex (Figure 4A, f) and naïve MDA5 is highly sensitive to trypsin digestion (Figure 5B, a), we hypothesized that MDA5 was released from the fibers through ATP hydrolysis, but even at the freely diffusing state, it retained a structure similar to that bound to dsRNA. To test this hypothesis, the fiber of poly(I:C) and MDA5 was isolated by SEC. The fiber was mixed with ATP and LGP2 to accelerate dissociation. The remaining fiber (pellet) and the released MDA5 (supernatant) were fractionated by ultracentrifugation. Native-PAGE (Figure 5E) revealed that the released MDA5 is composed of monomers and oligomers. Following trypsin digestion revealed that the released MDA5 exposed CARD1 (Figure 5F) as compared to naïve MDA5.

A single amino acid substitution in MDA5 (G821S) confers strongly increased constitutive activity, resulting in the constitutive production of IFN-I and inflammatory cytokines, and autoimmunity-like phenotype in mice (28). AFM observation of a single molecule revealed that wild type MDA5 adopts an open structure, composed of 3 to 4 subdomains and connecting linkers (Supplementary Figure S5B, left). However, G821S exhibited a closed, globular structure. This was further confirmed by AFM (Supplementary Figure S5B, right) and a high-speed AFM observation of a single molecule (Supplementary Figure S5C). Examination of these molecules by limited trypsin digestion revealed that the conformation of MDA5 and G821S were distinct, and CARDs were mostly masked in MDA5 but highly exposed in G821S (Supplementary Figure S5A, right). In summary, wild type MDA5 has an open structure with masked CARDs, whereas the overall structure of G821S is closed with a fully exposed CARDs. These observations prompted us to compare the molecular structure of MDA5 after its interaction with dsRNA and ATP or LGP2, by AFM (Figure 5G–I). As a result, MDA5 interacted with dsRNA, dissociated and then underwent major structural change from an open to closed conformation, in which the CARD1 was unmasked.

## DISCUSSION

### Proposed model for MDA5/LGP2 signaling

Naïve MDA5 is incompetent for signaling. For instance, cellular treatment of IFN-I induces MDA5 synthesis, but does not activate IRF-3 or NF-κB nor subsequent IFN-I genes. We hypothesize that naïve MDA5 is in inactive conformation, where CARDs are masked. The mechanism by which the MDA5 CARDs are sequestered in the naïve state is so far unknown. We believe the mechanism is different from RIG-I. One group describes crystal structure of MDA5 helicase-insert domain Hel2i which is, in RIG-I, region that interacts with CARDs keeping it inactive in the absence of RNA. In MDA5 this region is shorter. Moreover, in contrast to the RIG-I, there is no interaction between CARDs and CARD-deleted MDA5 (29). These differences suggest distinct mechanism of sequestration than that of RIG-I. In Figure S5A we showed that the naïve MDA5 does not expose CARDs: Band 3 (as a hallmark of closed CARDs) appears first and it is resistant to trypsin. This suggested that sequestration mechanism exists, possibly making a linker region between CARDs and helicase a good candidate for future investigation.

Based on the data presented above, we propose a mechanistic model by which LGP2 alters MDA5 signaling. Consistent with the previous report (23), LGP2 increases fiber formation of MDA5 along the length of long dsRNA. We found that LGP2 molecules are incorporated into the fibers. This periodic incorporation of LGP2 may facilitate elongation of the fiber by altering the conformation of growing termini.

It was previously reported that MDA5 fibers are dissociated through ATP hydrolysis (3,4). The ATP-dependent dissociation may be interpreted as a mechanism of negative regulation of the signaling. However, the addition of LGP2 and ATP to pre-formed MDA5 fibers promoted efficient fiber dissociation (Figure 4A), suggesting that the dissociation is related to the positive regulation of signaling.

The trigger of antiviral signaling by RLR was proposed to be exposure of the tandem CARD (C1 and C2, Figure 5A) through conformational change (1,3,22,29–31). To gain insights into structural changes, we used the limited digestion of MDA5 by trypsin. The naïve MDA5 molecule has an open structure and exhibited hypersensitivity to trypsin digestion. This is consistent with the AFM observation of a single MDA5 molecule (28). MDA5 in fibers undergoes a structural change and exposes its CARDs (Figure 5B). Upon ATP binding, as demonstrated in the presence of AMP-PNP, MDA5 undergoes a slight structural change where CARD1 is only partially exposed. Further addition of LGP2 promotes full exposure of the CARD1 and CTD. Whether the CARD2 is exposed remains to be elucidated. In the presence of ATP, which promotes fiber dissociation, the MDA5 conformation is identical to that within the fibers. This suggested that the released MDA5 did not adopt the naïve conformation and retains its ‘activated’ conformation with the exposed CARD1. This ‘memory’ model is consistent with the AFM observation of MDA5 structure (Figure 5G–I).

**Figure 6.**
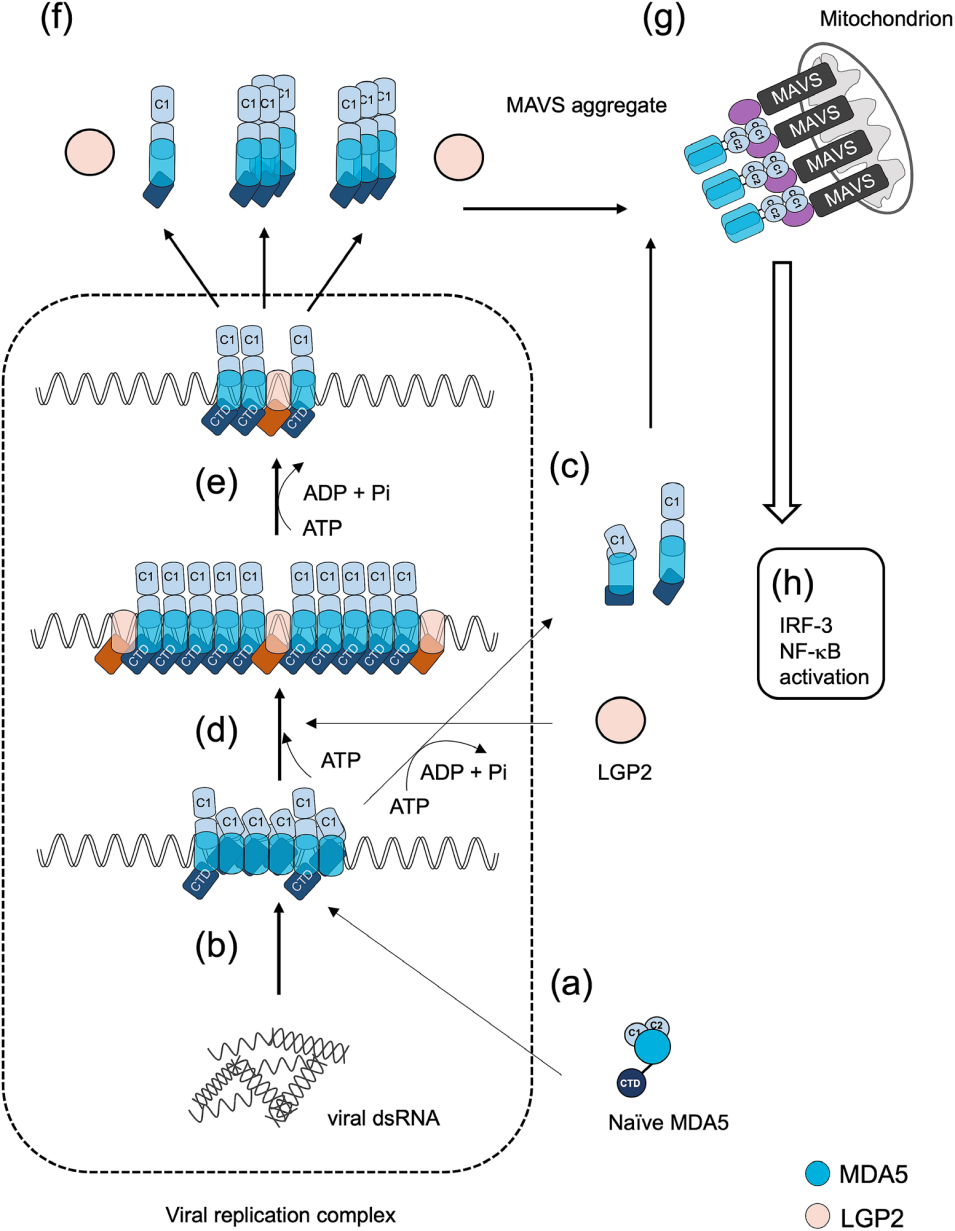
Model for activation of MDA5 by LGP2 and ATP. (**A**) Naïve MDA5 has an open structure and is hypersensitive to trypsin, however the tandem CARD is masked. (**B**) Upon binding with viral dsRNA localized within the viral replication complex, MDA5 forms fibers, in which it has a closed structure with partially exposed CARDs. (**C**) ATP hydrolysis weakly promotes MDA5 fiber dissociation. (**D**) In the presence of LGP2 and ATP, MDA5 further changes conformation to fully expose its CARD1. (**E**) Upon ATP hydrolysis, the fiber efficiently dissociates. (**F**) Released MDA5 retains its open structure as monomer/oligomer mixture and exits from the viral replication complex and migrates to its downstream adaptor, MAVS. (**G**) MDA5 forms a complex with MAVS on mitochondria. (**H**) Aggregation of MDA5 and MAVS results in the activation of transcription factors, including IRF-3 and NF-κB.

The conformational change of MDA5 through interaction with dsRNA, LGP and ATP is shown step-by-step in Figure 6. The activated MDA5 may migrate to downstream adaptor MAVS to form aggregates and trigger the signaling cascade, leading to the activation of IFN and IFN-stimulated genes.

In this report, we proposed a model of how LGP2 aids MDA5 to recognize long dsRNA. It was strongly suggested that ATP is essential for MDA5 fiber formation and production of free MDA5 in the activated conformation through its hydrolysis, where LGP2 contributed as a catalyst in these reactions, thus being the key positive regulator of the viral RNA recognition.

## Supplementary Material

gkaa935_Supplemental_File
