# Supplementary material for: Viral RNA recognition by LGP2 and MDA5, and activation of signaling through step-by-step conformational changes
Source: Nucleic Acids Res. 2020 Nov 2;48(20):11664–74. doi: 10.1093/nar/gkaa935 (PMC7672446; doi:10.1093/nar/gkaa935)
Supplement: gkaa935_Supplemental_File [file gkaa935_supplemental_file.pdf]

SUPPORTING INFORMATION

Figure S1. Antibody specificity and its labeling with Qdot

Recombinant MDA5 (FLAG-tagged) and LGP2 were immunoblotted with α-FLAG and α-LGP2 (top). A similar blot containing serially diluted LGP2 and MDA5 was visualized by Ponceau S staining, followed by probing with αLGP2-Qdot (bottom). LGP2-Qdot was detected by Typhoon 9410 imager.

Figure S2. Physiological concentration of MDA5 in cells

HeLa cells (10^6) were treated with IFN-β (1000 U/ml overnight) and whole cell lysate was prepared (2664 μg protein). Immunoblotting, using recombinant MDA5 as a standard, revealed that 10 μg (protein) lysate, contained 0.02 μg MDA5. Therefore, cellular content of MDA5 is 5.32 μg/10^6 cells. 10^6 cells contained 20 μl cytosol. Therefore, MDA5 concentration in cells is approximately 2.6 μM.

Figure S3. N-terminal sequencing of band 4 produced by limited trypsin digestion of MDA5

Band 4 generated by limited trypsin digestion (Figure 5A) was isolated and subjected to N-terminal sequencing by Edman degradation. The released amino acid by each cycle was analyzed by HPLC. Positions of amino acid are indicated as referenced by standards. The determined YMNPE sequence matched with amino acid sequence of human MDA5 starting from Y94. This is consistent with specificity of trypsin digestion (C-termini of R or K).

Figure S4. Trypsin digestion input samples

MDA5 or MDA/LGP2 alone or complexed with poly(I:C) (at 1:1 mass ratio) in the absence or presence of 2 mM AMP-PNP or 2 mM ATP were prepared as 25-μg samples. Before adding TPCK trypsin 10-μl samples were removed and subjected to SDS-PAGE, followed by silver staining using Sil-Best Stain One (Nacalai, Japan).

Figure S5. Trypsin digestion and AFM observation of recombinant MDA5 wt and MDA5 G821S

(A) Recombinant MDA5 and MDA5 G821S were digested with trypsin as described in Figure 5B, *a*, except with a lower trypsin concentration (TPCK trypsin 17 ng) and shorter time. (B) AFM analyses of MDA5 and MDA5 G821S. AFM images of MDA5 and MDA5 G821S. Left: 300 x 300-nm^2 images, Right: 75 x 75-nm^2 images. From these images, MDA5 monomers (objects with a total volume of 300±30 nm^3) were selected and quantified for diameter (bottom). (C) Single-molecule analysis of WT and G821S using high-speed AFM. Images are 62.5 x 62.5-nm^2 frames acquired at a rate of 1 frame per second. Schematic representation of each image is displayed below. Representative live images are shown. The minimum circum-diameter (MCD) of each frame is shown (bottom).

S1:

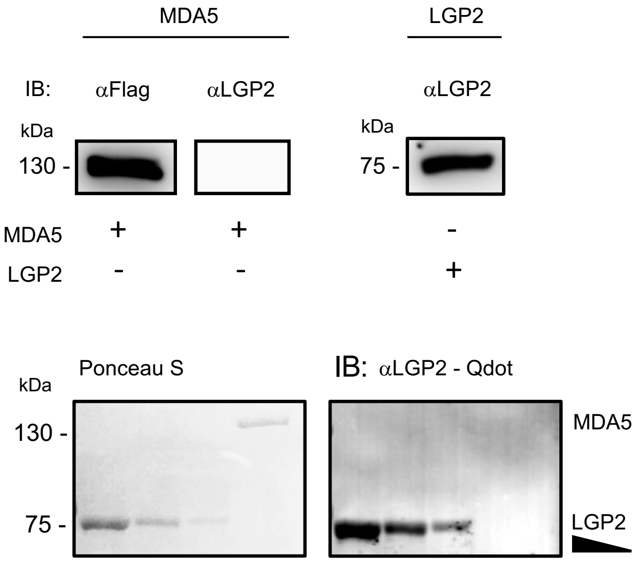
MDA5
LGP2
IB:
αFlag
αLGP2
αLGP2
kDa
130 -
kDa
75 -
MDA5 + + -
LGP2 - - +
Ponceau S
IB: αLGP2 - Qdot
kDa
130 -
75 -
MDA5
LGP2

S2:

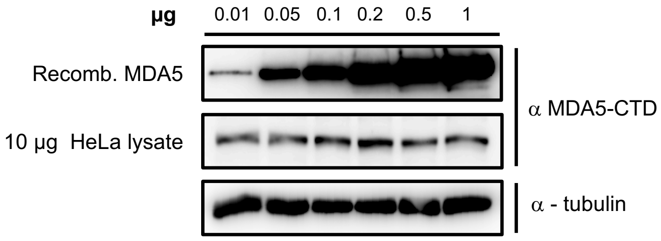
µg
0.01 0.05 0.1 0.2 0.5 1
Recomb. MDA5
10 µg HeLa lysate
α MDA5-CTD
α - tubulin

S3:

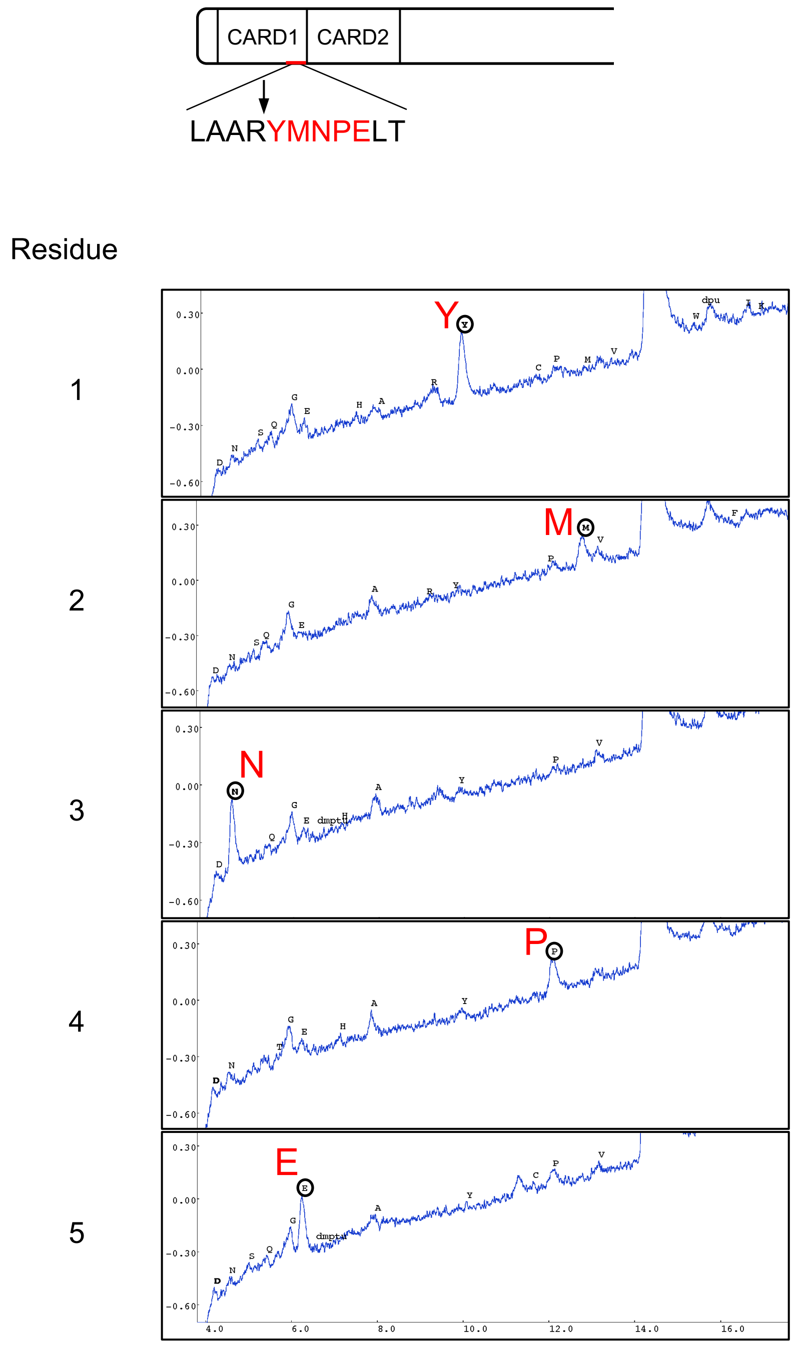

S4:

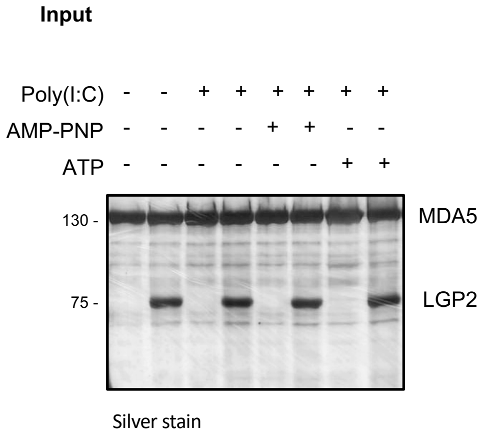

S5:

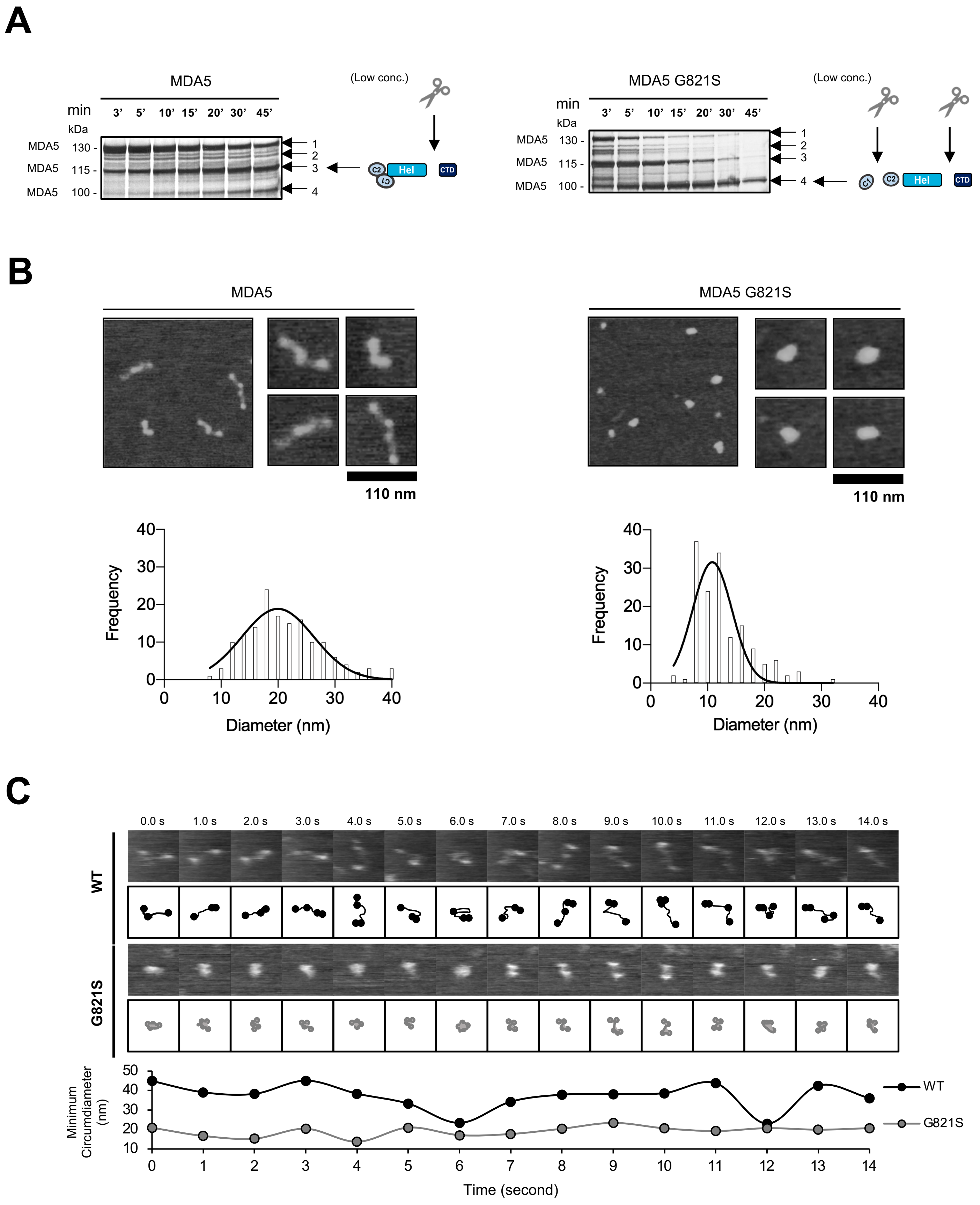
A
MDA5
(Low conc.)
min
3' 5' 10' 15' 20' 30' 45'
kDa
MDA5 130 -
MDA5 115 -
MDA5 100 -
1
2
3
4
C2
C1
Hel
CTD
MDA5 G821S
(Low conc.)
B
MDA5
MDA5 G821S
110 nm
110 nm
Frequency
Diameter (nm)
C
0.0 s 1.0 s 2.0 s 3.0 s 4.0 s 5.0 s 6.0 s 7.0 s 8.0 s 9.0 s 10.0 s 11.0 s 12.0 s 13.0 s 14.0 s
WT
G821S
Minimum Circumdiameter (nm)
Time (second)
WT
G821S
